# Echocardiogram-Guided Diagnosis of Anomalous Coronary Arteries: A Rare Presentation

**DOI:** 10.7759/cureus.39732

**Published:** 2023-05-30

**Authors:** Maya A Khatoun, Hassan Kanso, Teddy Gerges, Elie Chammas, Ali Msheik

**Affiliations:** 1 Cardiology, University of Balamand, Beirut, LBN; 2 Radiology, Clémenceau Medical Center, Beirut, LBN; 3 Anesthesia, Winchester Anesthesia Associates, Winchester, USA; 4 Cardiology, Clémenceau Medical Center, Beirut, LBN; 5 Neurological Surgery, Lebanese University Faculty of Medicine, Beirut, LBN

**Keywords:** asymptomatic, incidental radiological finding, anomalous coronaries, echography of the heart, coronary computed tomoangiography

## Abstract

Incidentaloma, a term that embodies the detection of certain problems during the performance of unreasonable investigations, resonates in the medical field. Retroaortic coronary sign is a recently recognized echocardiographic feature of the anomalous coronary artery. It is typically associated with anomalies of the left coronary artery, especially the left circumflex artery. As far as has been monitored, few echocardiographic signs that correlate with this feature have been identified. This feature often remains underdiagnosed on transthoracic echocardiograms due to confusion with artifacts, calcifications, and other cardiac structures. A 45-year-old male patient underwent regular cardiac routine assessment. Retroaortic anomalous coronary (RAC) sign was incidentally detected by transthoracic thoracic echocardiogram; consequently, the retroaortic route of the coronary artery was suspected. Coronary computed tomography angiography was requested to confirm the seen echocardiographic signs. After a 3D reconstruction imaging, the left circumflex retroaortic course was identified with right coronary sinus origin. This case ensures the importance of transthoracic echocardiography as a noninvasive tool in diagnosing anomalous coronary arteries. These anomalies are usually diagnosed by coronary computed tomography angiography and coronary angiography, mainly in the presence of retroaortic coronary sign or “crossed aorta sign.”

## Introduction

There is a variable morphology of the anatomy of the human coronary arteries. This morphology is considered anomalous once detected in lower than 1% of the population [1]. In normal cases, the left circumflex (LCX) originates from the left main artery, by which the latter originates in turn from the left coronary sinus [2]. One or two obtuse marginal branches arise from LCX after it enters the atrioventricular groove (AVG) [1]. Indeed, the left circumflex anomaly is one of the highest incidences among the congenital anomalies of the coronary artery, whether from the right coronary sinus (RCS) or the cusp (0.3-0.8%) [3]. It should be noted that the retroaortic route of an artery originates from the posterior course between the interatrial septum and non-coronary sinus. This route is also manifested by various forms of congenital coronary diseases, such as the ectopic origin of the left main artery and LCX artery [4]. Actually, this congenital anomaly is noticed through invasive coronary anomaly [5]. In 2017, Witt CM et al. shed light in their study on the relationship between anomalous coronary arteries and retroaortic coronary (RAC) sign on transthoracic echocardiography (TTE) [3]. The RAC sign can be seen on the four-chamber apical view as a tubular image above the mitral valve plan that is directed towards the RSV [6]. However, it might be confused with mitral annular calcification, calcified valves, or normal coronaries [3]. Particular echocardiographic signs have been detected on the parasternal long-axis view of TTE: “the Bleb sign,” which is an additional round structure under the non-coronary cusp sign [6]. It is considered that the retroaortic trajectory of an anomalous coronary is benign in the absence of any hemodynamic significance [4]. In this report, the case of a 45-year-old male with anomalous origin of LCX is described, primarily diagnosed by an incident on TTE through two echography signs, “Bleb and RAC,” and then confirmed by coronary computed tomography angiography (CCTA).

## Case presentation

**Figure 1:**
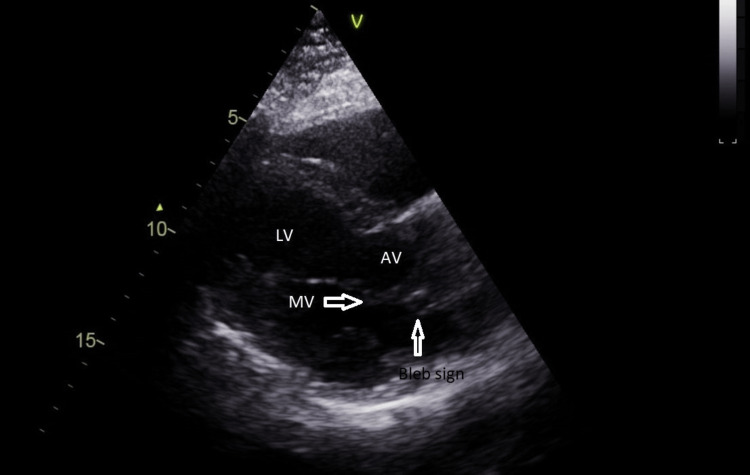
Conventional transthoracic parasternal long-axis view showing a small circle beneath the non-coronary cusp. Transthoracic bleb sign indicated by arrow. LV: Left ventricle; MV: Mitral valve; AV: Aortic valve.

A 45-year-old male patient was presented to the outpatient clinic for routine cardiac investigations. He is a non-smoker with no previous medical conditions or family history of cardiac diseases, and he practices high-intensity exercises regularly without any difficulties. Vital signs and BMI were within normal limits. The review of the cardiovascular system is negative for chest pain or dyspnea. In addition, the cardiovascular physical examination showed normal heart sounds, good peripheral body pulses and the electrocardiogram was also normal. A basic laboratory profile was ordered. Echocardiography and stress echocardiography were also done to the patient’s preference. Echocardiography showed normal left ventricular (LV) systolic function and no major valvular abnormalities, but a round structure under the non-coronary cusp (NCC) was manifested. Focusing on the short- and long-axis view of the heart, a linear structure across the aortic valve was seen, which led us to suspect the presence of anomalous coronary arteries with retroaortic course (Figure 1 and Video 1).

**Video 1:** Parasternal transthoracic short-axis view showing linear aspect across the aortic valve. Crossed aortic sign marked by an arrow, followed by the modified transthoracic four-chamber apical view, obtained by tilting the transducer to a more anterior plan. A binary structure above the mitral valve plane overlapping the aortic root was noted (the second arrow indicates RAC sign). (1006*700 dpi 300)

**Figure 2:**
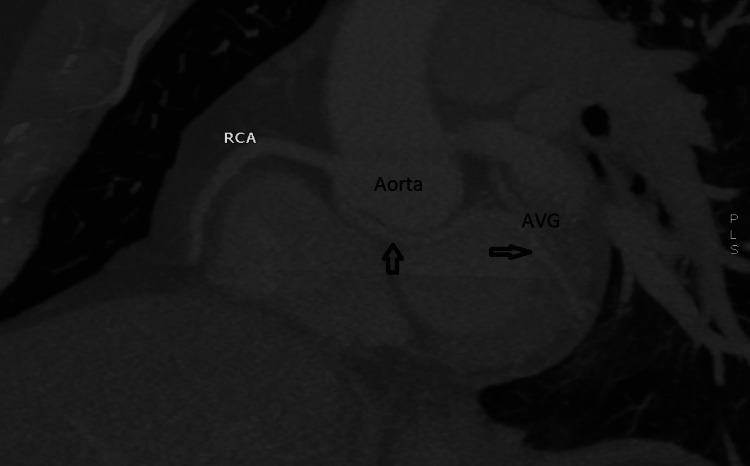
Cardiac CT with 3D reconstruction. The two black arrows indicate a retroaortic artery arising from RCS near the right coronary artery directed toward the atrioventricular groove. RCS: Right coronary sinus; RCA: Right coronary artery (925*489 DPI 300)

CCTA was demanded to confirm the diagnosis. The axial view revealed an unusual retroaortic artery arising from RCS near the right coronary artery and directed toward the AVG (Figure 2).

**Figure 3:**
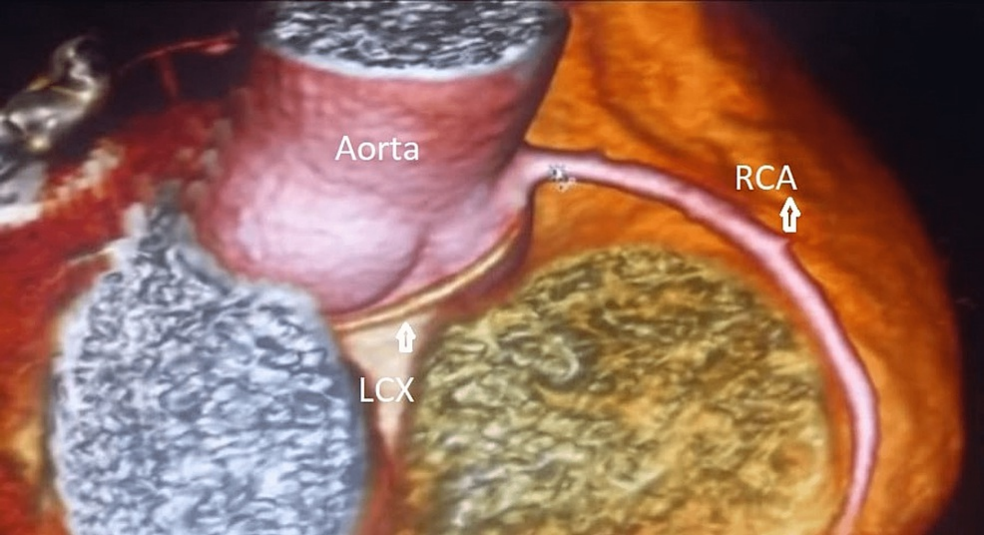
3D reconstruction cardiac CT. Left circumflex retroaortic artery arising near the right coronary artery with posterior trajectory (900*489 DPI:300). LCX: Left circumflex; RCA: Right coronary artery.

**Figure 4:**
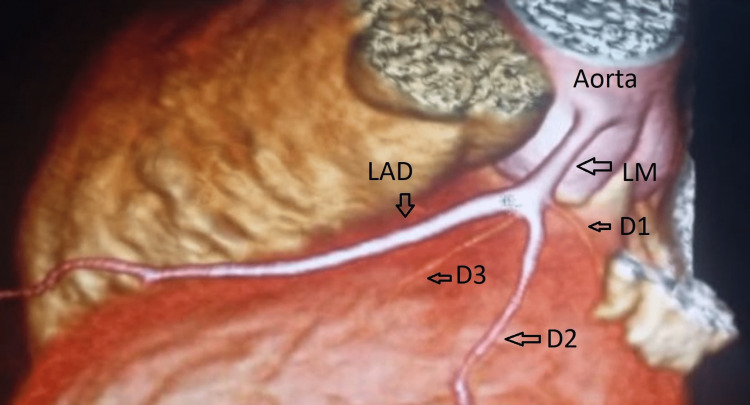
3D reconstruction cardiac CT image. The left main artery gives rise to one branch, which is the left anterior descending artery. LAD gives three diagonal branches (D1, D2, and D3). LAD: Left anterior descending; LM: Left main; D: Diagonal artery (1667*900 dimensions, DPI 300).

A retro aortic artery arising near the right coronary artery was detected after 3D reconstruction of images in the oblique view over the aortic area. The absence of left main bifurcation leads to the suspicion of LCX anomalous originating from the RSV (Figures 3-4).

Laboratory workup and stress echocardiography were completely normal, with excellent functional capacity and no inducible electric, echocardiographic, or clinical ischemia.

## Discussion

The LCX anomalous, arising from the RCS, is the most frequent anomaly of the congenital coronary anomalous artery (CCAA) [3]. Normally, it takes a path in the AVG in a clockwise direction to give marginal branches [1]. The retroaortic coronary artery is defined as a coronary artery coursing behind the aorta as a binary structure and crossing it perpendicularly in the long axis five-chamber view on TTE [5,6]. This is known as the RAC sign on TTE, one of the two signs described in our case. RAC sign is well linked with the anomalous of the left coronary artery [1]. In this sense, the Fisher test had significantly proved, with a p-value less than 0.001, the link between the RAC sign and retroaortic anomalous coronary artery [3]. Nonetheless, it should not be confused with coronary sinus, aortic valve calcifications, and artifacts [6]. In this patient, the tubular image was seen from different objections, making the artifact's diagnosis less likely. One of the artifacts' characteristics is the inconsistency in changing echo view [7]. Add to that, the coronary sinus is differentiated from retroaortic coronary by its location in the posterior plan, its large dimension, and its lower position than the AVG. On the other hand, the retroaortic coronary is characterized by its location in the anterior plan, its small dimension, and its passage by the atrioventricular sulcus, where LCX arises from the right coronary sinus [6]. The second discovered sign, in our case, is the Bleb sign. The bleb sign is usually visualized on the parasternal long axis of TTE. It is a hypoechoic round structure at the level of the aorta-mitral junction under the non-coronary cusp, similar to that found at the mid-esophageal aortic valve on the long axis of transesophageal echocardiogram [4]. Although it diagnoses the LCX anomalous artery, it is inconclusive [8]. It is difficult to be noticed and remains underdiagnosed because of its small size; this is in addition to the fact that it cannot be differentiated from mitral-aortic calcifications [6]. However, our patient is young, and the structure of his valves was normal. Despite being misinterpreted as a mitral-aortic abscess [6], the Bleb sign is distinguished from the latter by the absence of a clinical infectious picture in our case. Retroaortic course is discovered incidentally on coronary CT, MRI, and invasive coronary angiogram [4]. Though considered benign, it is associated with hemodynamic risks if accompanied by other reported features such as extrinsic compression due to coronary artery angulation, aortic root dilation, and an intramural course in the aortic wall. Surgical intervention is warranted if any compression is present from the pulmonary artery; otherwise, no medical therapy exists [2-5]. It is obvious that some reported cases in the literature are associated with adverse events [5]. Patients with this condition may have inducible ischemia that may cause angina, myocardial infarction, or even sudden cardiac death due to arrhythmias [3,5]. Our patient is asymptomatic with no inducible ischemia on stress echocardiography. Recognizing and never neglecting RAC and Bleb signs is essential to protect the coronaries in case of associated valve surgery [3]. We have confirmed the diagnosis without the need for an invasive procedure. While CCTA is considered the first recommended method to diagnose congenital coronary artery anomalies with high sensitivity, the RAC sign has high specificity, reaching 93.9% in one retrospective study. However, the sensitivity of the RAC sign is still not very high [3]. To our knowledge, no clear data was found about Bleb sign sensitivity and specificity. Indeed, further studies addressing the sensitivity and specificity of RAC and Bleb signs are required as the field of echocardiography has evolved.

## Conclusions

This case addresses the diagnosis of an anomalous left circumflex artery through two echocardiographic signs. Despite the massive revolution in the medical field, the RAC and Bleb signs have remained unrecognized, unnoticed, and misinterpreted echocardiographic signs. They have become strongly associated with the anomalous left circumflex artery. It is important to distinguish these signs from other similar findings in order to diagnose CCAA in its early stages. Nowadays, TTE is a cost-effective and non-invasive tool that plays a vital role in diagnosing anomalous coronary arteries through expert echocardiography specialists.
